# Disparities in Community Immunization Rates in New England: Findings From the Healthy Aging Data Reports

**DOI:** 10.1093/geroni/igaa057.253

**Published:** 2020-12-16

**Authors:** Alison Rataj, Nina Silverstein, Chae Man Lee, Haowei Wang, Richard Chunga, Taylor Jansen, Beth Dugan, Shuangshuang Wang

**Affiliations:** 1 University of Massachusetts Boston, Kingston, New Hampshire, United States; 2 University of Massachusetts Boston, Needham, Massachusetts, United States; 3 University of Massachusetts Boston, Boston, United States; 4 University of Massachusetts Boston, Boston, Massachusetts, United States; 5 Shandong University, Jinan, Shandong, China

## Abstract

Vaccinations are effective preventive tools yet are underutilized in the older adult population. The purpose of this study was to describe community rates of three vaccines (flu, pneumonia, and shingles) in MA, NH, and RI. State and community rates were compared to identify disparities. Data sources were the CMS Medicare Current Beneficiary Summary file (2013-2015) for the vaccine rates and the American Community Survey (2012-2016) for population characteristics. Small area estimation techniques were used to calculate age-sex adjusted community rates in each state and are reported at the community level and statewide. Results showed comparable rates of flu immunization (MA 60.8%; RI 59.1%; NH 59.3%). NH had the best statewide rate of pneumonia immunization (77.8%) and MA had the worst (72.0%). There was variation in shingles vaccination rate: 39.7% in MA and 30.3% in RI, perhaps reflecting differences in access. Within state disparities were observed. MA shingles vaccine rates ranged 57.80% - 21.17%, pneumonia ranged 79.78% - 61.21%, and flu ranged 71.09% - 51.46%. In RI shingles vaccination rates ranged 42.1% - 21.1%, pneumonia ranged 82.1% - 61.8% and flu ranged 68.4% - 52.1%. In NH pneumonia rates ranged 84.18% - 71.87% and flu ranged 67.14% - 52.11%. Strategies to address within and between state disparities are needed. Greater awareness of the benefits of preventive measures like vaccines may also help improve rates. Materials like the GSA guideline about aging and immunizations could be useful in educating providers and policymakers. This research is funded by the Tufts Health Plan Foundation.

